# *Naegleria fowleri* Meningoencephalitis Associated with Public Water Supply, Pakistan, 2014

**DOI:** 10.3201/eid2210.151236

**Published:** 2016-10

**Authors:** Najia K. Ghanchi, Erum Khan, Azam Khan, Wali Muhammad, Faisal Riaz Malik, Afia Zafar

**Affiliations:** Aga Khan University, Karachi, Pakistan (N.K. Ghanchi, E. Khan, F.R. Malik, A. Zafar);; Karachi Water and Sewerage Board, Karachi (A. Khan);; World Health Organization, Karachi (W. Muhammad)

**Keywords:** *Naegleria fowleri*, meningoencephalitis, water supply, brain diseases, vector-borne infections, ameba, Pakistan, parasites

**To the Editor:** *Naegleria fowleri*, a free-living ameba, causes acute, fulminant, fatal primary amebic meningoencephalitis (PAM) in persons with history of recreational activities in warm freshwater (*1**,**2*). During 2008–2009, thirteen case-patients with PAM and no history of recreational water activity were reported from Karachi, Pakistan (*3*). Since then, PAM caused by domestic water exposure, nasal cleansing by using neti pots, and ablution has been reported globally (*4**–**6*). During 2014–2015, the Aga Khan University Hospital clinical laboratory in Karachi confirmed 19 PAM case-patients without history of recreational activities in warm freshwater. 

Karachi has a subtropical, arid climate and long summers (March–October). The increasing number of PAM cases might be attributable to rising environmental temperatures and a dysfunctional water supply system in Karachi (*7*). Data indicating direct evidence of *N. fowleri* amebae in Karachi’s water supply are limited, but consistent annual reemergence of PAM in patients without history of recreational water exposure raises concerns about Karachi’s water supply.

In August 2014, a previously healthy 34-year-old man living in Karachi and having no recreational water exposure was admitted to the Aga Khan University Hospital with multiple episodes of vomiting, severe headache, and fever. Cerebrospinal fluid culture showed a low glucose level (46 mg/dL [reference 45–80 mg/dL]) and high levels of protein (216 mg/dL [reference 20–40 mg/dL]), erythrocytes (30 cells/mm^3^ [reference 0–10 cells/mm^3^]), and leukocytes (1,440 cells/mm^3^ [reference 0–5 cells/mm^3^]; 65% lymphocytes and 35% neutrophils). PCR confirmed presence of *N. fowleri*. The patient died 4 days after admission, and cerebrospinal fluid and blood cultures were negative for bacterial and fungal growth.

We investigated for presence of *N. fowleri* amebae in domestic water and for the patient’s possible exposure. In September 2014, we collected 23 samples from 2 water treatment plants (plants A and B), their pumping stations, and catchment areas (Technical Appendix Figure). Plant A supplied 13 of the samples from its water distribution system, which provided water to the patient’s residence and neighborhood mosque (Table). The other 10 samples were from Plant B and its water distribution system (Technical Appendix Table), for which the local government had initiated additional chlorine enhancement because of previously occurring PAM cases. Plant A had no chlorine enhancement. Real-time PCR, as described (*8*), confirmed presence of *N. fowleri* amebae in the plant’s water supply distribution. Both plants are monitored for quality control by using World Health Organization water treatment procedures guidelines (http://apps.who.int/iris/bitstream/10665/44584/1/9789241548151_eng.pdf). The plants’ water distribution exit points had the highest residual chlorine levels (0.5 ppm) (*9*), and levels gradually decreased beyond the plants. Residual chlorine was undetectable in plant A’s water distribution to the patient’s residential area (12 km from the plant); however, with plant B’s additional chlorine enhancement stations, chlorine levels were detectable in all households tested.

**Table:** Characteristics of 13 water samples collected from water treatment plant A and its distribution system, including water supplied to the apartment and neighborhood mosque of a patient with primary amebic meningitis, Karachi, Pakistan, 2014*

Water supply	Sample location	Sample type	Total chlorine, mg/L	Temperature, °C†	Culture positivity for FLAs	PCR results for *Naegleria fowleri *amebae‡	Distance relationships of water samples
Reservoir§	Water from Kinjhar	Untreated	ND	30	++	–	From reservoir to plant A, ≈100 km
Water treatment plant A	Filtration unit¶	Treatment underway	<0.5	29.5	++	–	
	Plant A exit point	Filtered and chlorinated	0.5	30	++	–	
Pumping station#	Pumping station, site 1	Filtered and chlorinated	ND	30	++	–	From plant A to pumping station, ≈11 km
	Pumping station, site 2	Filtered and chlorinated	ND	30	++	–	
Catchment areas: patient’s apartment and neighborhood	Mainline	Filtered and chlorinated	ND	30	–	–	From pumping station to patient’s house, 1km
	Underground boring well water**	Untreated	ND	28.5	+	–	Within patient’s house, ≈10 m
	Underground tank	Mixed††	ND	28	+	–	Within patient’s house, ≈10 m
	Overhead tank	Mixed	ND	31	++	+	Within patient’s house, ≈10 m
	In-house storage tank‡‡	Mixed	ND	30	++	–	Within patient’s house, ≈5 m
	Bathroom	Mixed	ND	29	+	–	Within patient’s house, ≈5 m
	Neighborhood mosque	Filtered and chlorinated	ND	31	++	+	From patient’s house to mosque, ≈100 m

*FLAs, free-living amebae; ND, not detected; ++, >3 amebae seen with 40x magnification; +, 1–3 amebae seen with 40 x magnification; –, no amebae detected. †Water temperatures of 25°C–40°C are conductive for flourishing of *Naegleria fowleri* amebae. ‡PCR was negative for other pathogenic FLAs such as *Balamuthia* or *Acanthamoeba* spp. §Kinjhar Lake, located in Sindh province, Pakistan, is the main reservoir that supplies water to Karachi.  ¶The filtration unit was the only site for which 2 samples were taken; only 1 sample was taken from all other specific sample locations.  #Samples were taken from a single pumping station at 2 separate sites. **Underground wells provide additional water supplies locally known as boring wells. ††Water from these wells is not treated and is mixed with treated water from the main water supply in storage tanks. ‡‡Underground and overhead tanks are shared by >100 households, so for continuous water supply, residents keep small tanks in their homes.

Residual chlorine and specimen positivity for free-living amebae were inversely correlated. Differences were most noticeable in samples collected from plant A’s distributed water, compared with plant B’s water distribution samples. PCR confirmed *N. fowleri* amebae in 2 water samples collected from the patient’s household overhead storage tanks and neighborhood mosque. The samples, taken from plant A’s distributed water, showed no residual chlorine and a temperature >30°C. Lack of detectable chlorine and water temperature >25ºC might have provided favorable conditions for *N. fowleri* amebae to thrive in domestic water (*5*); water temperatures 25°C –40°C are favorable for *N. fowleri* growth. Absence of the amebae in plant B’s water suggests the importance of enhanced chlorine pumping at distribution points beyond water treatment plants for maintain residual chlorine in Karachi’s domestic water supply.

Because water supply can be intermittent, underground and overhead storage tanks are essential for Karachi homes. To ensure continuous domestic supply, water is stored in overhead tanks and pumped from tanks into homes as needed. Water storage in tanks perhaps facilitated propagation of *N. fowleri* amebae in domestic and mosque water. During the summer, ambient temperatures reach 44°C, leading to increased water temperatures in overhead tanks. We found water temperatures up to 34°C, which may facilitate excystation of *N. fowleri* amebae to infective forms. Slime, dirt, and high ambient temperatures likely explain *N. fowleri* multiplication in storage tanks, the possible source of infection for this patient in Karachi.

Presence of *N. fowleri* amebae in mosque water is alarming. Ablution (Wudhu) is a ritual performed by Muslims before offering prayers and involves thorough cleaning of mouth, ears, face, arms, feet, and nasal passages, the latter by inhaling water forcefully up the nostrils. Performing this activity with contaminated water could be a communal source for potential outbreaks.

Karachi water supply authorities have initiated chlorine enhancement at various sites beyond plant B, and our findings support the need for this enhancement. We recommend that the government implement measures to maintain appropriate chlorine levels in the domestic water supply and at recreational sites and to develop effective amebae-monitoring programs. The public should use boiled or filtered water for nasal cleansing, regularly clean storage tanks, and add supplemental chlorine to water in homes, especially during the summer.

Technical Appendix Details of water treatment plant B’s water samples and locations of plants A and B, their distribution systems, water sampling sites, and other sites related to samples collected in Karachi, Pakistan, 2014.
