# Supplementary material for: Naegleria fowleri Meningoencephalitis Associated with Public Water Supply, Pakistan, 2014
Source: Emerg Infect Dis. 2016 Oct;22(10):1835–7. doi: 10.3201/eid2210.151236 (PMC5038392; doi:10.3201/eid2210.151236)
Supplement: Technical Appendix — Details of water treatment plant B’s water samples and locations of plants A and B, their distribution systems, water sampling sites, and other sites related to samples collected in Karachi, Pakistan, 2014. [file 15-1236-Techapp-s1.pdf]

Article DOI: http://dx.doi.org/10.3201/eid2210.151236

Naegleria fowleri Meningoencephalitis Associated with Public Water Supply, Pakistan

Technical Appendix

Technical Appendix Table. Water samples (n = 10) collected from water treatment plant B and its distribution system, including households, Karachi, Pakistan, 2014*

Water supply	Characteristic							
	Sample location	Sample type	No. samples	Total chlorine, mg/L	Temperature, °C†	Culture positivity for FLAs	PCR results for *Naegleria fowleri* amebae‡	Distance relationships of water samples
Reservoir§	Water from Kinjhar	Untreated	2	ND<br>NT	26<br>27	++<br>++	–<br>–	From reservoir to plant B, 100 km
Water treatment plant B	Filtration unit	Treatment underway	2	1	28	+	–	
	Plant B exit point	Filtered and chlorinated	1	1	24	–	–	
Pumping station	Pumping station 1	Filtered and chlorinated	1	0.5	27	+	–	From plant B to pumping station, 17.5 km
	Pumping station 2	Filtered and chlorinated	1	>2.5	26	–	–	From pumping station site 1 to pumping station site 2, 3.5 km
Catchment areas: consumer households	Household 1, underground storage tank	Filtered and chlorinated	1	0.5	29	+	–	From pumping station site 2 to household 1, 2.03 km
	Household 1, overhead storage tank	Filtered and chlorinated	1	<0.5	34	+	–	
	Household 2, underground storage tank	Filtered and chlorinated	1	traces	30	–	–	From pumping station site 2 to household 2, 0.5 km

*FLAs, free-living amebae; ND, not detected; NT, not tested; +, 1–3 amebae seen with 40 x magnification; ++, >3 amebae seen with 40 x magnification; –, no amebae detected.
†Water temperatures of 25°C–40°C are conductive for flourishing of *Naegleria fowleri* amebae.
‡PCR was negative for other pathogenic FLAs such as *Balamuthia* or *Acanthamoeba* species.
§Kinjhar Lake, located in Sindh province, Pakistan, is the main reservoir that supplies water to Karachi.

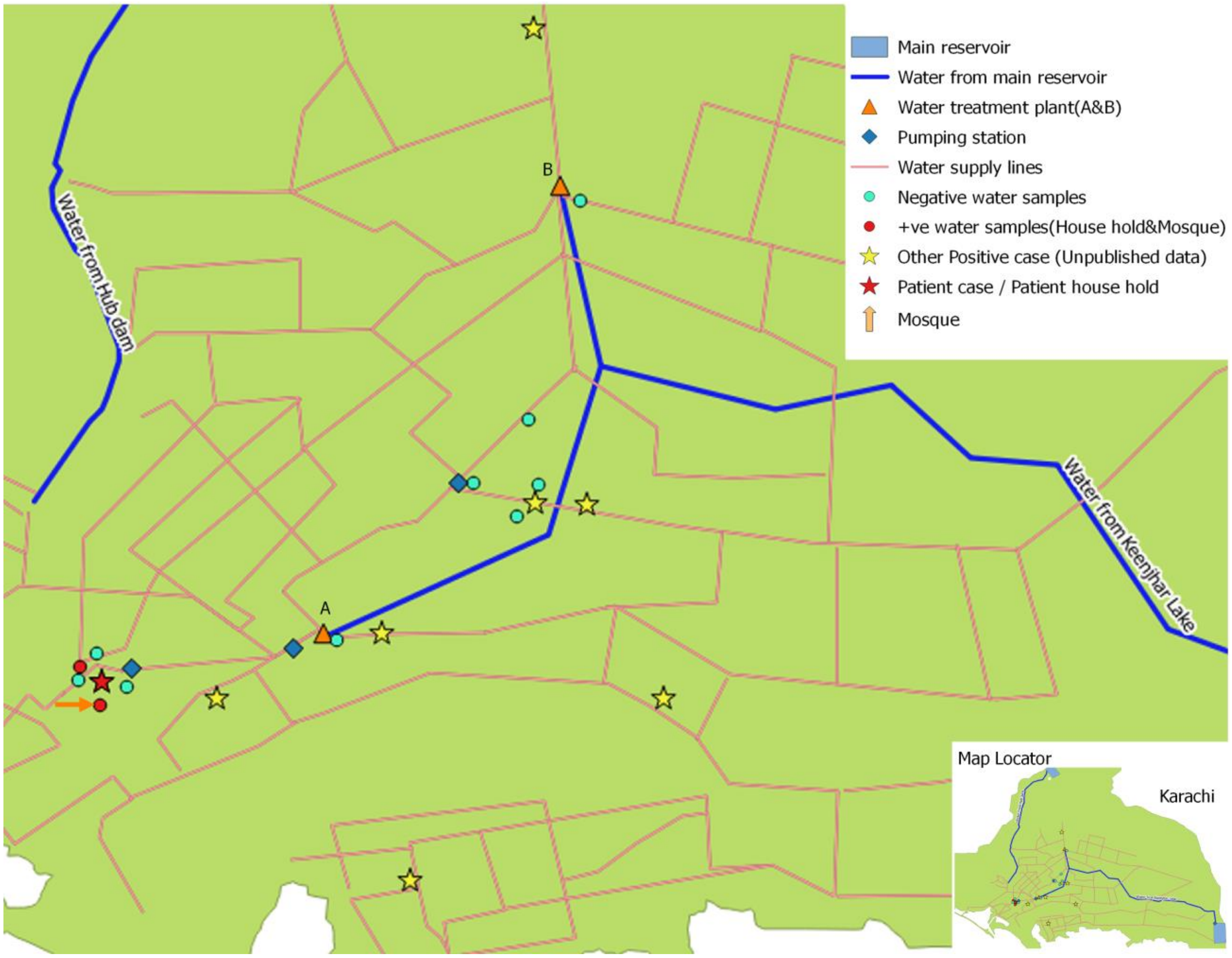

Technical Appendix Figure. Locations of water treatment plants A and B and their distribution systems, water sampling sites, and other sites related to samples collected in Karachi, Pakistan, 2014. Inset shows main reservoir and its distance from sampling sites (≈100 km from Karachi). Because of a high number of cases reported before the case in this study, chlorine enhancement was initiated at water treatment plant B but not at plant A. *N. fowleri* was not detected after chlorine enhancement at plant B's distribution system in this study.
